# Dermal Penetration Studies of Potential Phenolic Compounds Ex Vivo and Their Antioxidant Activity In Vitro

**DOI:** 10.3390/plants11151901

**Published:** 2022-07-22

**Authors:** Aurita Butkeviciute, Kristina Ramanauskiene, Vaida Kurapkiene, Valdimaras Janulis

**Affiliations:** 1 Department of Pharmacognosy, Lithuanian University of Health Sciences, Sukileliu Ave. 13, LT-50162 Kaunas, Lithuania; valdimaras.janulis@lsmuni.lt; 2 Department of Clinical Pharmacy, Lithuanian University of Health Sciences, Sukileliu Ave. 13, LT-50162 Kaunas, Lithuania; kristina.ramanauskiene@lsmuni.lt (K.R.); vaida.kurapkiene@lsmu.lt (V.K.)

**Keywords:** antioxidant, apple, dermis, epidermis, topical formulation, penetration

## Abstract

Phenolic compounds with miscellaneous biological activities are an interesting component in dermatology and cosmetology practices. The aim of our study was to determine the phenolic compounds released from emulsion, emulgel, gel, ointment, and oleogel formulations penetration into human skin layers, both the epidermis and dermis, and estimate their antioxidant activity. The ex vivo penetration study was performed using Bronaugh type flow-through diffusion cells. Penetration studies revealed that, within 24 h, the chlorogenic acid released from the oleogel penetrated into skin layers to a depth of 2.0 ± 0.1 µg/mL in the epidermis and 1.5 ± 0.07 µg/mL in the dermis. The oleogel-released complex of phenolic compounds penetrating into epidermis showed the strongest DPPH free radical scavenging activity (281.8 ± 14.1 µM TE/L). The study estimated a strong positive correlation (r = 0.729) between the amount of quercetin penetrated into epidermis and the antioxidant activity detected in the epidermis extract. Plant based phenolic compounds demonstrated antioxidant activity and showed great permeability properties through the skin.

## 1. Introduction

Currently, there is an increased interest in natural products that contain components obtained from plants. Natural products are abundant, and almost 35,000–70,000 plants species have been screened to date for their biological effects [1]. Phenolic compounds, with antioxidant activities, are one of the main substances formed by the secondary metabolism of plants [2]. These biologically active compounds can be found in an immense diversity of matrices, such as fruits, vegetables, and others [2]. The apple is one of the most cultivated fruits in the world, with high nutritional value [3]. In the human diet, apples are an important constituent, and their nutritional values are determined by phenolic compounds [4,5]. Phenolic acids and flavonoids with anti-inflammatory, antioxidant, and immune system-promoting effects are potentially valuable in wound healing therapies, for the prevention of chronic inflammation of the skin and skin disorders caused by viral, bacterial, or fungal infections, premature aging, hyperpigmentation, and for skin protection from UV radiation [6,7,8]. The potency of phenolic compounds as plant-derived natural agents to act as photoprotectors, antioxidants, and antimicrobial substances are of interest for cosmetic and therapeutic purposes.

In cosmetology and dermatology, a wide range skin care products using plant extracts have been developed [9]. The skin is a strong and flexible organ with barrier properties which play a relevant role in protecting the body from destructive exposure to external and internal environments [10,11]. Active substance permeation across the skin is a complex process. and the main steps are the diffusion of the substance within the topical formulation, the release from it, and the penetration into the skin. Because of the complex and non-homogeneous skin structure, the description and modelling of active substance permeation are quite difficult [12]. The challenge concerning the bioavailability of externally applied active substances is composed of and estimated by many factors, including biological (age, skin location and condition, cardiovascular functions, and metabolism), physicochemical (active substances properties, such as molecular weight, size, spatial structure of the molecule, and lipophilicity), and delivery system characteristics [10,13,14]. The formulations have to be chemically, physically, and microbiologically stable to assure the stability and deliverability of active substances to the target skin layers [15]. Therefore, besides the stability of the active agents, their skin absorption, that is, the permeation of their potent antioxidant compounds through the epidermis into the dermis, is a prerequisite for efficiency.

The application of topical preparations depends on the type and difficulty of the disease. For skin disorders, the topical route is most preferred. A topical drug delivery system can be defined as the direct application of a formulation containing active substances to the skin [16]. The topical preparation has several benefits, such as the ability to introduce the active agents to a required location, bypassing the gastrointestinal tract [17]. In addition, local deliveries ensure increased bioavailability, the bypassing of first-pass metabolism, and consistent delivery for an extended time [18,19]. One aspect of the combined process of penetration via the skin is the release of the content of active ingredients from the formulation. The faster the release rate, the earlier the substance can access the skin and permeate into it [9]. As a part of the fruit matrix, phenolics are not fully released, and the released compounds are poorly absorbed. The bioavailability of phenolic compounds is not particularly high [20]. Previous studies have exposed that different groups of phenolics are absorbed at a rate of 0.3–43.0%, and that the metabolite levels circulating in the plasma can be low [21]. There is information suggesting that the route of administration, the form of dosage release, and absorption are known to affect bioavailability [22]. The phenolic compound’s bioavailability depends on the subclass of phenolic compounds and their physicochemical properties, including the degree of polymerization, glycosylation, or molecular properties, their polarity and their interaction with nutrients, the proteins and carbohydrates in their cells, as well as the other components of the formulation in which they are introduced [13,23]. Prognosticating the penetrability of a desired agent through the skin is, in general, not easy because of the greatly complex nature of the structures and mechanisms that make up the delivery pathway [24].

According to the scientific literature, until recently, in vitro studies have been carried out, evaluating the release of active compounds from semi-solid pharmaceutical forms through synthetic membranes. The results of our study showed that the released amounts of phenolic compounds depend on the chosen carrier. Because of their safe and effective use, it is important to investigate the penetration of phenolic compounds in more detail by determining their penetration into the skin layers. Skin penetration studies should also be conducted in a timely manner, as most cosmetic and therapeutic agents are dominated by naturally occurring active compounds. At present, much attention is paid to skin protective agents; therefore, plant extracts are often present in the compositions as a source of antioxidants. In this study, it was chosen to investigate the penetration of the active compounds of apple extract into the skin, due to its increasing application in the production of cosmetics. In this experiment, it has been chosen to produce semi-solid pharmaceutical formulations that have been used for a long time in ointments and emulsions, and which are increasingly being used in gel and emulgel due to their positive properties. It has also been chosen to produce oleogel, which is a great alternative to petroleum-based ointments due to its superior sensory properties. The results of this research will enable the development and production of new, innovative, semi-solid pharmaceutical forms and cosmetic products using a complex of phenolic compounds.

The aim of our study was to determine the penetration of phenolic compounds released from emulsion, emulgel, gel, ointment, and oleogel formulations into human skin layers, both the epidermis and dermis, and to estimate their antioxidant activity.

## 2. Results and Discussion

### 2.1. Quantitative Profile of Identified Phenolic Compounds of Apple Extract

**Figure 1:**
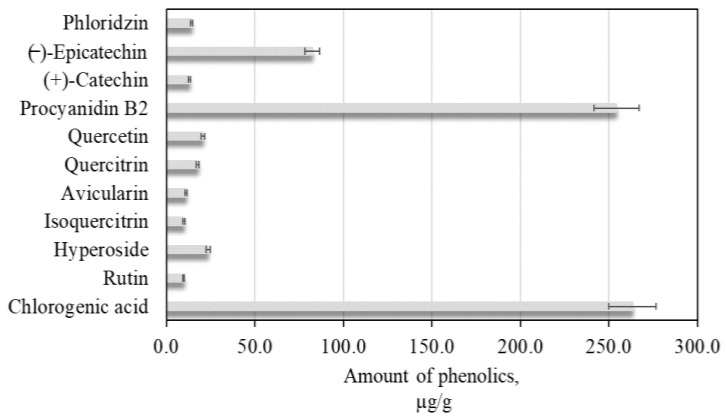
Content of phenolic compounds of apple extract.

The apple extract of cultivar Kostele, grown in Lithuanian climatic conditions, was used in the study. During the first stage of the research, we determine variations in the qualitative and quantitative composition of the phenolic compounds of apple extract. In apple extract, we identified and quantified: flavonols (rutin, hyperoside, isoquercitrin, avicularin, quercitrin, and quercetin), flavan-3-ols (procyanidin B2, (+)-catechin, and (−)-epicatechin,), a dihydrochalcone (phlorizin), and a phenolic acid (chlorogenic acid). During the study, five series of apple lyophilized were used, and the study was repeated three times, the results of which are presented in Figure 1. The total amount of phenolic compounds determined in the sample of the apples from the Kostele cultivar was 720.3 ± 36.0 µg/g. Compounds of the flavan-3-ol group predominated among all the identified phenolics. The total amount of the biological active compounds of this group (364.2 ± 18.2 µg/g) accounted for 50.6% of all the phenolic compounds identified. Jakobek et al. determined that the flavan-3-ol amount in fruit samples of apple cultivars grown in Croatian orchards varied from 20.0 µg/g to 690.0 µg/g [25]. Procyanidin B2 was the prevailing compound among flavan-3-ols in the apples from the cultivar selected for this study (Figure 1). Wojdyło et al., analyzing in Poland, cultivated apple extract’s phenolic compound profiles and found that levels of procyanidin B2 varied from 70.0 to 2000.0 µg/g [26].

The total content of flavonols identified and quantified in the apple samples was 92.8 ± 4.6 µg/g, which accounted for 12.9% of the total phenolics detected (Figure 1). The flavonols identified and quantified in the apple samples can be ranked by their content in the following ascending order: rutin < isoquercitrin < avicularin < quercitrin < quercetin < hyperoside (Figure 1). Hyperoside was the predominant compound among flavonols in the apple samples. Belviso et al. found that the level of hyperoside in apple samples grown in Italian orchards ranged from 0.3 µg/g to 2.0 µg/g [27]. In our study, the apple samples contained higher contents of hyperoside compared to those found in fruit samples of apple cultivars grown in Italian orchards.

The content of the identified and quantified chlorogenic acid was 263.3 ± 13.6 µg/g, which accounted for 36.6% of the total phenolic compounds detected (Figure 1). According to the scientific literature, chlorogenic acid may constitute 21.0–90.0% of the total amount of phenolic compounds in apples [28]. Rana et al. established that the content of chlorogenic acid in apples ranges from 106.8 µg/g to 198.9 µg/g [29]. The dihydrochalcone group compound phlorizin was also found in the apple samples. Its amount in the apple sample was 14.2 ± 0.7 µg/g, accounting for only 2.0% of the total phenolics identified in the apple sample (Figure 1). Piccolo et al. established that the amount of phlorizin in apples varies from 10.0 µg/g to 50.0 µg/g, confirming the results of our study [30].

After determining the qualitative and quantitative composition of phenolic compounds in apple extracts of the cultivar grown in Lithuania, the aim was to evaluate whether apple extract can be used in topical preparations and how the carrier influences the release and penetration of active compounds into the skin layers.

### 2.2. Ex Vivo Test of the Skin Permeation of Individual Phenolic Compounds

Penetration of active substances across the skin is a more complex process than their release from the delivery system, especially because of the heterogeneous of the skin tissue. The skin penetrability barrier is comprised of three different layers: *Stratum Corneum*, viable epidermis, and dermis [9]. The viable epidermis is an avascular matrix, containing mainly of keratinocytes and consisting of about 40.0% protein, 40.0% water, and 15.0–20.0% lipids [24]. The dermis environment includes the blood vessels, lymphatics, and nervous system within the skin, as well as the various skin appendages (sweat glands, sebaceous glands, and hair follicles), and this skin layer mainly consists of fibroblasts responsible for the synthesis of collagen, elastin, and glycosaminoglycans [31].

**Figure 2:**
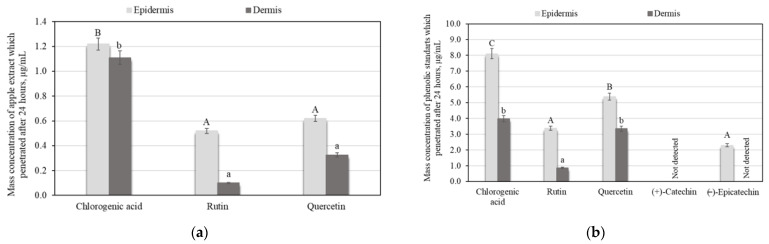
Results of penetration study: (**a**) Penetration of apple extract into skin layers after application for 24 h; (**b**) Penetration of chlorogenic acid, rutin, quercetin, (+)-catechin, and (−)-epicatechin standard solutions into skin layers after application for 24 h. Uppercase letters indicate a statistically significant difference in the amount of phenolic compounds that penetrated into the epidermis layer; lowercase letters indicate a statistically significant difference in the amount of phenolic compounds that penetrated into the dermis layer (*p* < 0.05).

HPLC analysis showed that, of the eleven phenolic compounds determined in apple extract, only three compounds of apple extract penetrated into the skin layers after 24 h, namely chlorogenic acid, rutin, and quercetin (Figure 2a). Statistically significantly, the most penetrating phenolic compound of apple extract was chlorogenic acid, with penetration levels of 1.2 ± 0.06 µg/mL in the epidermis and 1.1 ± 0.05 µg/mL in the dermis (Figure 2a). According to the results of the studies, we did not find a significant difference between rutin and quercetin penetration into the epidermis and dermis (Figure 2a).

To confirm the penetration of phenolic compounds into the dermis, we prepared solutions of purified individual phenolic compounds and evaluated their penetration into the skin layers after 24 h. Assessing the skin penetration of purified individual phenolic compounds, we found that (+)-catechin did not penetrate either the epidermis or the dermis within 24 h (Figure 2b). Chlorogenic acid penetrated into the skin layers, at a level of 8.1 ± 0.4 µg/mL in the epidermis and −4.0 ± 0.2 µg/mL in the dermis (Figure 2b). No significant difference between the penetration of chlorogenic acid and quercetin into the dermis was found. The least (0.9 ± 0.04 µg/mL) penetrated compound into the dermis was rutin (Figure 2b). The results of the distribution of biologically active compounds in the skin revealed that there is no statistically significant difference between the penetration of rutin and (−)-epicatechin into the epidermis (Figure 2b).

Because of the barrier effect of the skin, most of the active agents delivered topically on the skin have a low natural perviousness [13]. The challenges involved in the penetration process are skin location, anatomy, age, skin hydration, and the physicochemical characteristics of the compositions [14]. Penetration of external substances is not directly connected to the thickness of the skin at the particular site. Others factors, such as the number of follicles, the thickness of the *Stratum Corneum*, and the sebum composition, as well as the distance between capillaries and the surface of the skin, all appear to be of influence [10]. Previous studies have reported that the penetration rates depend on the molecular weight and polarity. The smallest and the most hydrophilic compounds exhibited the highest penetration rates [9]. In permeation tests of (−)-epigallocatechin gallate (EGCG) and quercetin from green tea and *Gingko biloba* via the excised human skin [32], the generality of quercetin was determined in the viable epidermis, but the level of EGCG in the *Stratum Corneum* was higher than the contents in the viable epidermis and in the dermis. Abla et al. analyzed the delivery of phenolic compounds with various polarity from the propylene glycol vehicle into porcine ear skin. Phenolic compounds, which are more polar ((+)-catechin, resveratrol, and curcumin), were mostly concentrated in the *Stratum Corneum*, whereas less-polar retinol concentrated in the underlying layers of the skin [33]. Alonso et al. studied phenolic compounds ((−)-epicatechin, resveratrol, quercetin, rutin) and Trolox. These compounds were readily absorbed by the skin by means of an in vitro percutaneous experiment. The results indicate that these compounds, with a hydrophobic character, were located in the outermost layers of the skin (the *Stratum Corneum* and viable epidermal layer) [34]. In vitro penetration research using guinea pig skin and Yucatan micropig skin, lipophilic resveratrol, with its smaller molecular weight, was mostly allocated in the dermis, meanwhile hydrophilic chlorogenic acid was mainly found in the epidermis [35]. In vitro testing has revealed that caffeic and chlorogenic acid permeate the *Stratum Corneum* faster than glycoside oraposide, and this study showed that the aglycones of phenolic compounds exhibit significantly better permeation potency than their glycoside forms [36]. In our ex vivo study, catechins group compounds did not penetrate into the skin, or showed only slight penetration potency. Perhaps the results of such research were influenced by the high molecular weight of the catechins and their binding to the lipids of the skin, which reduced or stopped catechin penetration [37]. Catechins are highly reactive substances which can be lost by oxidative degradation or interaction with skin proteins such as collagen, reducing the penetration of these compounds into the skin layers [38]. A previous study showed the hydrolysis by skin esterase as a potential degradation mechanism of catechin group compounds in the skin [39].

An ex vivo penetration study of purified individual phenolic compounds confirms the results determined by the study of apple extract, showing that chlorogenic acid, rutin, and quercetin penetrate into both skin layers, the epidermis and the dermis. Studies of the penetration of apple extract, with a complex of phenolic compounds, into the skin show that although the extract has a multicomponent matrix, not all active compounds will penetrate into the skin layers, or will be able to solve skin problems. In the next stage of the study, we selected dermally active compounds, namely chlorogenic acid, rutin, and quercetin, to evaluate how different delivery systems influence active compounds penetration into the skin layers.

### 2.3. Ex Vivo Test of the Skin Permeation of Individual Phenolic Compounds from Semi-Solid Forms

At this stage of the study, we evaluated how the active compounds released from the semi-solid pharmaceutical formulations penetrated into the skin layers. Five different delivery systems were selected for the study: emulsion, emulgel, gel, ointment, and oleogel. The composition of the formulations is shown in Table 1. All of the modeled pharmaceutical formulations had a pH value ranging from 5.8 to 6.3 at room temperature. The gel formulation had the highest pH value (6.3), while the emulsion formulation had the lowest value (5.8). We evaluated the viscosity of five pharmaceutical forms with different lipophilicity properties. We estimated that the more hydrophobic base formulations, for example, ointment and oleogel, had a highest viscosity >11 Pa·s and 10.7 Pa·s, respectively. Meanwhile, the more hydrophilic formulation, in our case, emulsion, had the lowest viscosity of 4.3 Pa·s. In our experiment, all produced semi-solid formulations had a slightly yellow color and a homogeneous, acceptable texture, without any visible particles, and had a base odor at room temperature. All of the semi-solid forms containing apple extract and mixture of individual phenolic compounds had low pH values, which ensured their non-irritating effect and safety for use on the skin.

**Figure 3:**
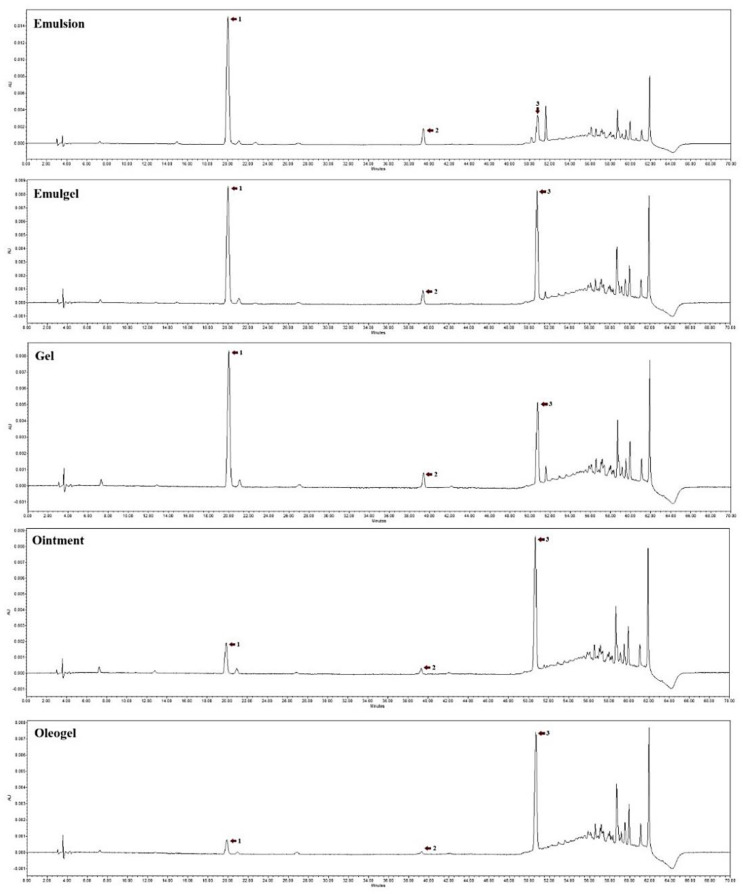
HPLC chromatogram of individual phenolic compounds from semi-solid forms. Analytes established at λ = 320 nm, and λ = 360 nm. 1—chlorogenic acid; 2—rutin; 3—quercetin.

The evaluation of the penetration of mixture solutions of individual phenolic compounds incorporated into five different pharmaceutical forms showed that only chlorogenic acid, rutin, and quercetin were released from the experimental formulations; the chromatographic profiles are shown in Figure 3.

**Figure 4:**
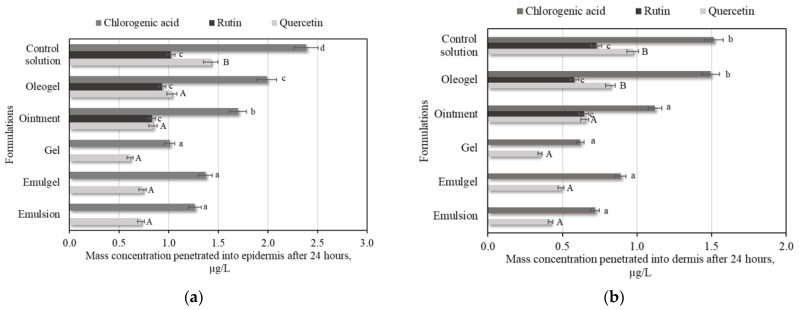
Results of penetration study: (**a**) Permeation of the mixture of chlorogenic acid, rutin, quercetin, (+)-catechin, and (−)-epicatechin standard solution from semi-solid forms into the epidermis after application for 24 h; (**b**) Permeation of the mixture of chlorogenic acid, rutin, quercetin, (+)-catechin, and (−)-epicatechin standard solution from semi-solid forms into the dermis after application for 24 h. Uppercase and lowercase letters indicate statistically significant differences between the amount of phenolic compounds that penetrated into the skin layers from semi-solid pharmaceutical forms (*p* < 0.05).

In the ex vivo penetration studies, we found that after 24 h analysis, the individual phenolic compounds and apple extract’s phenolic compounds penetrated into the skin layers, so in the next step, we sought to determine whether different delivery systems would affect the penetration of phenolic compounds into the skin layers. A control solution of a mixture of individual phenolic compounds was used in the study to compare the penetration of the active compounds into the skin from the solution and from the semi-solid pharmaceutical forms. Penetration studies revealed that, within 24 h, the chlorogenic acid released from the oleogel formulation penetrated into skin, the level of penetration into the epidermis was 2.0 ± 0.1 µg/mL, and the level of penetration into the dermis was 1.5 ± 0.07 µg/mL (Figure 4). The level (0.8 ± 0.05 µg/mL) of quercetin penetrated into the dermis from the oleogel formulation differed significantly from the amounts released from the emulsion, emulgel, gel, and ointment formulations (Figure 4b). We found that, in emulsion, emulgel, and gel formulations, inserted rutin did not penetrate either the epidermis or dermis (Figure 4). However, rutin released from ointment and oleogel formulations penetration into epidermis and dermis (Figure 4). The penetration studies of phenolic compounds from semi-solid pharmaceutical formulations revealed that the delivery system may affect the penetration of individual phenolic compounds into the skin layers.

Permeation into the skin from topical preparations may be influenced, not only by the properties of active substances, but also by the delivery system characteristics and the active compounds interactions with the components of the formulations. Therefore, it is important to choose a suitable base to act as a carrier for the active substances. Hydrophobic formulations using vegetable oils and liquid paraffin as ingredients may penetrate into the first upper layers of the *Stratum Corneum* [40], but under the influence of the systematic application of the hydrophobic formulations, lipophilic components of a base may be incorporated into the lipids of the *Stratum Corneum* [41]. Lipophilic bases, especially Vaseline petroleum jelly, predict prolonged contact of the formulation with the skin, as they tend to remain on the skin surface [41]. Oleogels were determined to enhance both skin retention and permeation of many active agents [42,43].

Hydrophilic bases usually provide high rates of release in vitro, as they are easily permeated by the acceptor fluid [44]. Emulsion bases affect the skin barrier and thus, have a relevant effect on the skin delivery of active agents. Emulsions may improve the rate of the *Stratum Corneum* hydration, and this occurs by the direct contact of the external aqueous phase of hydrophilic emulsion with the skin [45]. Hydrophobic emulsions have a similar effect as that of hydrophobic bases, indirectly improving the rate of the *Stratum Corneum* hydration, thanks to their occlusive properties [41]. The rate of skin permeation and retention reached with hydrophilic bases may be lower than that provided by emulsion or hydrophobic bases. Emulsifiers used in emulsion bases can permeate into the *Stratum Corneum* lipids and act as permeation enhancers, changing the *Stratum Corneum* lipid composition, as well as increasing the solubility of active agents within the *Stratum Corneum* lipids [46,47,48]. Previous studies have shown that with the application of emulsions with lower oil levels, in comparison with emulsions with higher oil ratios, the contents of phenolic compounds were lower in the *Stratum Corneum*, whereas higher amounts have been determined in the epidermis and dermis [12]. One of the reasons for this action could be an increased phenolic compound diffusion due to the lower viscosity of the emulsions with lower oil concentrations, as they tend to improve the release and penetration rates. Another reason could be a hyperhydration of the skin due to the application of formulations with higher water levels. Under normal conditions, the *Stratum Corneum* is relatively dry, in which water amounts reach about 20.0%, and its enhanced hydration leads to a reorganization of lipid lamellar structures and generally increases the delivery success of topical preparations [12].

The correct selection of components of semi-solid forms, for example, oils, emulsifiers, or hydrophobic solvents for the active agent plays a relevant role in the evolution of topical formulations providing efficient delivery of active substances to the skin [41]. Previous studies have revealed that the incorporation of chlorogenic acid, resveratrol, curcumin, and quercetin to oil in water microemulsions, containing sucrose laurate or di-2-ethylhexyl sodium sulfosuccinate, could increase the delivery of these phenolic compounds into the dermis, when compared with the microemulsions containing Tween 80 [35,49]. Bertges et al. showed that, in study using a gel cream formulation, which is usually used as a vehicle in commercial products, this was not adequate for the delivery of the biological active compounds to the skin, since it did not induce the permeation of the phenolic compounds in the epidermis [50]. In contrast, Bolzinger et al. showed greater penetration of chlorogenic acid from microemulsion than from the gels or emulsions [51].

Properly selected semi-solid forms, and their ingredients, may affect the penetration of phenolic compounds into the skin layers, both the epidermis and dermis. Depending on the intended use of the product, it is important to select appropriate topical formulations, which can ensure the penetration of the substances into the desired skin layer and ensure the pharmacological action of the biologically active compounds. In ex vivo dermal penetration studies, we found that the choice of a delivery system may affect the release and dermal penetration of active compounds. In our studies, the release and penetration of chlorogenic acid and quercetin into the skin layers were not affected by the delivery system as carrier, and these compounds penetrated into both skin layers, the epidermis and dermis. However, the delivery system affected rutin skin penetration. Rutin penetrates into the skin layers only from oleogel and ointment formulations. The results of our study show that the penetration of phenolic compounds into the deeper layers of the skin may depends on the delivery system used.

### 2.4. Antioxidant Activity In Vitro

The human skin is continually and directly affected by stressful environmental factors such as UV radiation or pollution, both of which trigger the generation of reactive free radicals. Free radicals are identified by one or more unpaired electrons and are able to enter into destructive chemical bonds with proteins of the skin, provoking chemical and functional changes in the skin matrix [52]. Free radicals also attack and react with the skin cell molecules in the dermis, resulting in wrinkles due to the cross-linking of collagen and elastin [53]. For prevention of oxidative stress and to enhance the reparation of the DNA, antioxidants should be applied topically.

**Figure 5:**
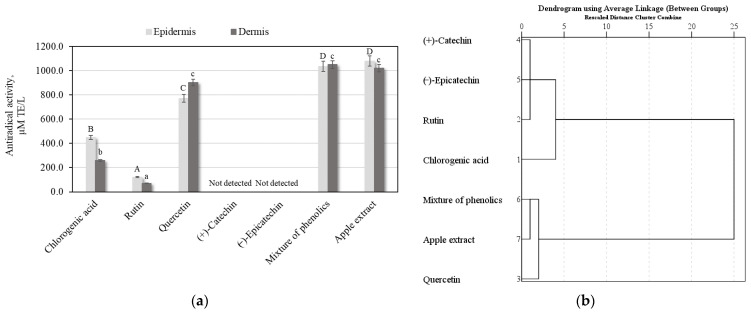
Results of antioxidant activity studies: (**a**) Antiradical activities of human skin extracts after applying the pure individual phenolics, mixture of phenolic compounds, and apple extract; (**b**) The dendrogram illustrates variation in the antioxidant activities of human skin extracts after applying experimental samples. Uppercase letters indicate a statistically significant difference in the antiradical activities of human skin epidermis extracts; lowercase letters indicate a statistically significant difference in the antiradical activities of human skin dermis extracts (*p* < 0.05).

In our study, we determined the antioxidant potency of methanolic human skin extracts using the DPPH free radical scavenging method after applying the pure individual phenolic compounds, the mixture of phenolic compounds, and apple extract. The negative control group was a blank skin sample that showed no antioxidant activity. We found that the antiradical activities of epidermis extracts ranged from 120.7 ± 6.03 µM TE/L to 1080.1 ± 54.0 µM TE/L, and those of dermis extracts ranged from 71.0 ± 3.5 µM TE/L to 1050.8 ± 52.3 µM TE/L (Figure 5a). The strongest antiradical activity (1080.1 ± 54.0 µM TE/L) was determined in the epidermis extract after the application of the apple extract. The weakest antiradical activity was determined in the skin extract after the application of rutin, whose DPPH free radical scavenging activities were 120.7 ± 6.03 µM TE/L and 71.0 ± 3.5 µM in the epidermis and dermis extracts, respectively (Figure 5a). Studies have shown that quercetin penetrates the skin, mostly into the dermis, and maintains strong antioxidant potency (Figure 5a). Alonso et al. described that the high antioxidant potency of methanolic porcine skin extract determined by the DPPH free radical scavenging method after applying agents with a high antioxidant potential (rutin, quercetin, and Trolox). Results of these studies showed a high penetration of some antioxidants into the skin [34].

The antioxidant activities of human skin extracts after applying the pure individual phenolic compounds, mixture of phenolic compounds, and apple extract after 24 h was divided into 5 clusters (Figure 5b). The phenolic compounds (+)-catechin and (−)-epicatechin were assigned to cluster I, in which antioxidant activity was not detected. Such results may have been due to the fact that the compounds of the catechin group did not penetrate into the skin layers. Rutin was assigned to cluster II, in which the lowest antiradical activity was determined. Chlorogenic acid was assigned to cluster III, which had average antioxidant potency. The mixture solution of individual phenolic compounds, as well as and apple extract, were assigned to cluster IV, in which the strongest antioxidant activity was established. Quercetin was assigned to cluster V, which had stronger antioxidant activity compared to average antioxidant activity (Figure 5b). The results of the study revealed that human skin extracts, after applying the mixture of phenolic compounds and apple extracts, showed stronger antioxidant activities compared to the activities of extracts after the application of individual phenolic compounds.

**Figure 6:**
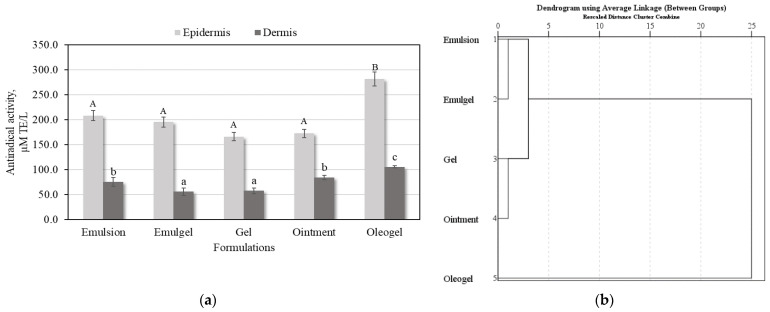
Results of antioxidant activity studies: (**a**) Antiradical activities of human skin extracts after applying semi-solid formulations with a mixture of phenolic compounds solution; (**b**) The dendrogram illustrates variation in the antioxidant activities of human skin extracts after applying semi-solid formulations with a mixture of phenolic compounds solution. Uppercase and lowercase letters indicate a statistically significant difference in the antiradical activities between semi-solid formulations (*p* < 0.05).

Antioxidant activities of human skin extracts after applying five different semi-solid formulations with individual phenolic compound complexes was performed using the DPPH method. The strongest antiradical activity resulted from the phenolic compound complex penetrating into the epidermis, and the antioxidant activity of epidermis extracts ranged from 166.2 ± 8.3 µM TE/L to 281.8 ± 14.1 µM TE/L (Figure 6a). The oleogel-released complex of phenolic compounds penetrating into epidermis showed the strongest DPPH free radical scavenging activity (281.8 ± 14.1 µM TE/L); meanwhile the gel formulation-released phenolic compounds showed the weakest antiradical activity (166.2 ± 8.3 µM TE/L) (Figure 6a). The oleogel formulation-released phenolic compounds penetrated into dermis revealed the strongest antiradical activity (105.6 ± 2.1 µM TE/L), while the weakest DPPH free radical scavenging activity (56.0 ± 2.8 µM TE/L) was observed in the phenolic compounds released from emulgel formulation (Figure 6a).

The antiradical activities of human skin extracts after applying semi-solid formulations after 24 h were divided into 3 clusters (Figure 6b). Phenolic compounds from emulsion and emulgel penetration into skin layers were assigned to cluster I, in which average antioxidant activities was determined. Phenolic compounds from gel and ointment permeation into dermal layers were assigned to cluster II, which had lowest antioxidant activities. Phenolic compounds from oleogel penetration into the epidermis and dermis were assigned to cluster III, in which the strongest antioxidant activity was detected (Figure 6b).

Phenolic compounds act as an important source of natural antioxidants. The modern viewpoint regarding UV protection strategies favor the UV filter effect, or the topical application of antioxidant agents [54]. The phenolic compounds can absorb UV radiation due to the presence of chromophores in their molecular structure. Therefore, they obviate UV radiation penetrate into the skin. This property improves the solar protection of the topical product and neutralizes the harmful effects of oxidative stress after sun exposure [55]. A previous study showed the sun protection factors of phenolic compounds, established at the smallest (SPF from 2 to 12) and medium (SPF from 12 to 30) levels of protection [9]. Consequently, the possibility of precluding or diminishing UV-induced photodamages proceed from the plant phenolic compounds into important topical preparation components. Petruk et al. described the quercetin inhibition of UV-induced inflammation in primary human keratinocytes and protection of mice skin from UV radiation-induced damage [56]. Potapovich et al. reported that the post-treatment of normal human epidermal keratinocytes (NHEKs) after UV exposition with plant quercetin, resveratrol, and verbascoside was efficient at eliminating the overproduction of peroxides and inflammatory mediators [57]. Moreover, the pretreatment of epidermal cells with phenolic agents, such as resveratrol and quercetin, reduces free radical formation and prevents DNA damage [55]. According to the scientific literature, free radicals can lead to early aging, since reactive oxygen species can connect with proteins such as collagenase, elastase, and tyrosinase of the skin, resulting in the degradation of collagen and elastin [2]. Cherubim et al. determined the anti-collagenase and anti-elastase effects of phenolic compounds, especially quercetin [55]. In a study performed on human volunteers, the topical application of quercetin and its derivative showed positive effects in regard to elasticity, moisturization, and depth of wrinkles [58]. Consequently, plants generate phenolic compounds to protect themselves from solar radiations, and these secondary metabolites can be used as natural antioxidants able to protect the skin from photo-aging. These active agents can protect the skin by absorbing UV radiations, inhibiting free radical reactions induced by UV in cells, and modulating antioxidant and inflammatory systems [56].

Topical preparations enriched with phenolic compounds may also have wound-healing properties. Wound healing is a dynamic process including complex interactions between cellular, molecular, biochemical, and physiological effects, which result in the regeneration and replacement of injured connective tissue at the wound site [59]. Keratinocytes, the most prevalent cell type in the epidermis, and fibroblasts, the predominant cell type in the dermis, have important roles in the process of skin repair after injury, and their interactions are critical for this process. Keratinocytes and dermal fibroblasts interact closely with one another, and they require cell–cell interactions to produce a cellular environment conducive to wound repair [60]. Previous studies have begun to investigate mechanisms by which fibroblast dysfunction could contribute to pathological wound healing states [61]. A study on the topical application of chlorogenic acid showed that it can accelerate the process of excision wound healing through its antioxidant activity and its significant ability to increase collagen synthesis and take part in different phases of the wound healing process [62]. Flavonoids, such as quercetin, are powerful antioxidants that have an important wound healing property. Salehi et al. described that quercetin and its derivates reduced wound area and increased wound contraction [58].

In our study, we determined the strength of the correlation between the amounts of individual compounds penetrated into the skin layers and the antioxidant activity detected in these skin layers. The Pearson correlation coefficients are shown in Table 2.

The study estimated a strong positive correlation (r = 0.729) between the amount of quercetin penetrated into epidermis and the antioxidant activity detected in the epidermis extract. Moderate positive correlations, r = 0.688 and r = 0.500, were determined between the amounts of chlorogenic acid and rutin penetrated into epidermis and the antioxidant activity determined in these extracts (Table 2). All individual phenolic compounds, namely chlorogenic acid, rutin, and quercetin, showed a strong positive correlation between the content of these compounds penetrated into the dermis and the antioxidant activity found in the dermis extracts (Table 2).

The cosmetic and dermatological interest of phenolic agents is mainly based on antioxidant potency. The application of antioxidants in topical products decreases oxidative damage, presenting a great alternative for the therapy and prevention of premature aging. It also provides photoprotective activity and helps with the treatment of sensitive or sun-stressed skin by its anti-inflammatory effect.

## 3. Materials and Methods

### 3.1. Plant Materials

The apple cultivar Kostele was used in this study. The apple trees were grown in the experimental orchard of the Institute of Horticulture, Lithuanian Research Centre for Agriculture and Forestry, Babtai, Lithuania (55°60′ N, 23°48′ E). The altitude of Babtai is 57 m above sea level. The apples harvested in September 2021 were immediately lyophilized and used for the study.

### 3.2. Chemicals and Solvents

All solvents, reagents, and standards used were of analytical grade. The standards used in the HPLC analysis were the following: hyperoside, rutin, quercitrin, phlorizin, procyanidin B2, and chlorogenic acid, obtained from Extrasynthese (Genay, France); (+)-catechin and (–)-epicatechin, purchased from Sigma-Aldrich GmbH (Buchs, Switzerland), and avicularin and isoquercitrin, obtained from Chromadex (Santa Ana, CA, USA). The chemicals applied in the modeling of semi-solid pharmaceutical forms were methanol, glycerin, olive oil, sorbitan monolaurate (Span 20), polyethylene glycol sorbitan monolaurate (Tween 20), Vaseline, sodium chloride obtained from Sigma-Aldrich Chemie GmbH (Steinheim, Germany). Poloxamer 407 was obtained from Fagron (St. Paul, MN, USA). PIONIER^®^ PLW was obtained from Hansen & Rosenthal KG^®^ (Hamburg, Germany). Ethanol from AB Stumbras (Kaunas, Lithuania). Purified deionized water used in the tests was prepared with the Milli-Q^®^ (Millipore, Bedford, MA, USA) water purification system. The reagents used in the antioxidant activity assay were 6-hydroxy-2,5,7,8-tetramethylchroman-2-carboxylic acid (Trolox), and 2,2-diphenyl-1-picrylhydrazyl (DPPH) acquired from Scharlau (Barcelona, Spain).

### 3.3. Preparation of the 1.0% Ethanolic Apple Extract 

The 1.0 g of the lyophilized apple sample was weighed and mixed with 100.0 mL of the 70.0% (*v*/*v*) ethanol until the apple samples were completely dissolved. The received extract was filtered through a paper filter, and the residue on the filter was washed with 70.0% (*v*/*v*) ethanol in a 100 mL flask until the accurate volume was achieved.

### 3.4. Preparation of the 1.0% Ethanolic Mixture of Phenolic Compounds 

The 1.0 g of individual phenolic compound standards: chlorogenic acid, rutin, quercetin, (+)-catechin, and (–)-epicatechin was weighed and mixed with 100.0 mL of the 70.0% (*v*/*v*) ethanol until the compound was completely dissolved. The 0.2 mL from each individual compound (chlorogenic acid, rutin, quercetin, (+)-catechin, and (–)-epicatechin) solution was weighed and mixed in a ratio 1:1:1:1:1.

### 3.5. HPLC-PDA Analysis for the Establishment of Phenolic Compounds

The qualitative and quantitative composition of phenolic compounds in the experimental samples were determined by using the HPLC-PDA method described by Liaudanskas et al. [64].

### 3.6. Preparation of Semi-Solid Formulations

All the experimental formulations compositions are shown in Table 1. Gel was prepared using poloxamer 407. The appropriate content of the poloxamer was weighed (18.0% (*w*/*v*)) and mixed with the appropriate content of purified water, and the mixtures were left in a refrigerator (5 °C) for 24 h until dissolved and homogeneous gel forms were obtained. A total of 10.0 g of glycerin, 10.0 g of olive oil, 6.0 g of Span 20, 6.0 g of Tween 20, and water ad 100.0 ± 0.5 g were mixed with a magnetic stirrer IKA^®^ C-MAG HS 7 (IKA^®^-Werke GmbH & Co. KG, Staufen Im Breisgau, Germany) until homogeneous emulsion forms were obtained. Equal amounts of gel (10.0 g) and emulsion (10.0 g) were mixed with a magnetic stirrer until an emulgel of homogeneous structure was obtained. Oleogel was modeled using PIONIER^®^ PLW basis. Vaseline was the basis for the ointment. All the experimental formulations were stored in a refrigerator (at 5 °C).

### 3.7. Ex Vivo Skin Penetration Study

Studies to determine the penetration of phenolic compounds into the human skin ex vivo were approved by the Kaunas Region Bioethical Committee (corresponding bioethical permission approval number: BE-2-41). Skin samples were received with informed consent from female patients (ages 25–40) undergoing elective abdominoplasty in the Department of Plastic and Reconstructive Surgery, Hospital of Lithuanian University of Health Sciences, Kaunas Clinics. Any extraneous subcutaneous fat was removed from the dermal surface. The skin was frozen and stored at −20 °C for not longer than 6 months before use. Ex vivo skin penetration experiment was slightly modified and performed using the methodology described by Zilius et al. [65]. Studies were performed using Bronaugh type flow-through diffusion cells with full-thickness human skin. The efficient diffusion area in the cells was 0.64 cm^2^. The diffusion cells were placed on a metallic heating block, maintaining 37.0 ± 0.5 °C temperature using a Grant TC120 thermostated circulating water bath (Grant Instruments Ltd., Cambridge, UK). The acceptor medium (0.9% NaCl solution) was circulated underneath the skin samples, maintaining 0.6 mL/min of circulation rate using a Masterflex L/S peristaltic pump with multichannel pump head (Cole-Parmer Instrument Co., Vernon Hills, IL, USA). The infinite dose of experimental formulations was applied on the outer human skin side surface, and the diffusion cells were covered with aluminum foil. After 24 h, tested formulations were removed from the human skin surface. The skin samples (0.64 cm^2^) were trimmed off, removing the outer residuals. The epidermis was separated from the dermis by applying the dry heat separation method [66], and the samples were separately extracted with methanol under 30 min sonication.

In order to evaluate the penetration of the phenolic compounds of apple extract through the skin, 1.0 mL (1.0%) of apple extract was applied to the skin. In the next step, we chose to add 1.0 mL (1.0%) mixture solution of purified individual phenolic compounds to each prepared semi-solid formulation.

### 3.8. Evaluation of Antioxidant Activity

The DPPH free radical scavenging activity was estimated by applying the method proposed by Brand-Williams et al. [67]. The antioxidant activity was assessed by in vitro spectrophotometric assay using a spectrophotometer (Spectronic CamSpec M550, Garforth, UK). The DPPH solution in 96.3% (*v*/*v*) ethanol (3.0 mL, 6 × 10^−5^ M) was mixed with 10 μL of the experimental sample. A decrease in absorbance was measured after 30 min at the 517 nm wavelength. The negative control group was a blank skin sample that showed no antioxidant activity.

### 3.9. Data Analysis

The statistical analysis of the research data was performed by using Microsoft Office Excel 2013 (Microsoft, Redmond, WA, USA) and SPSS 25.0 (SPSS Inc., Chicago, IL, USA) computer software. All the results were presented as the means of the results of three consecutive tests and their standard deviations. To estimate the variance in the quantitative composition, we calculated the coefficient of variation. ANOVA was used to determine that the differences between the compared data were statistically significant. If the variances of independent variables were estimated to be equal, Tukey’s multiple comparison test was applied. The differences were evaluated as statistically significant at *p* < 0.05.

## 4. Conclusions

The evaluation of pure individual phenolic compounds of apple extract penetration into the skin layers was performed. Only three compounds of apple extract penetrated into the skin layers, namely chlorogenic acid, rutin, and quercetin. Based on the results of this study, chlorogenic acid penetrated into the skin layers, including the epidermis and dermis. Ex vivo penetration studies revealed that, within 24 h, the chlorogenic acid released from the oleogel formulation penetrated into the skin layers. In emulsion, emulgel, and gel, the inserted rutin did not penetrate either the epidermis or the dermis, but in ointment and oleogel, inserted rutin penetrated into both skin layers. The results of our study show that the penetration of phenolic compounds into the deeper layers of the skin may depend on the delivery system used. The antioxidant potency of human skin extracts using the DPPH method after applying the pure individual phenolic compounds, apple extract, and five different semi-solid formulations were determined. The results of the study revealed that the oleogel-released complex of phenolic compounds penetrating into epidermis showed the strongest DPPH free radical scavenging activity. The study estimated a strong positive correlation (r = 0.729) between the amount of quercetin penetrated into epidermis and the antioxidant activity detected in the epidermis extract.

Plant based phenolic compounds demonstrated antioxidant activity and showed great permeability properties through the skin. Due to their natural origin and weak toxicity, phenolic compounds are an interesting agent for innovative pharmaceutical treatments for skin disorders or the development of new cosmetic products.

## Figures and Tables

**Table 1:** Composition of the delivery systems.

Composition	Emulsion	Emulgel	Gel	Ointment	Oleogel
Poloxamer 407, g	-	Mixed emulsion and gel 1:1	18.0	-	-
Glycerin, g	10.0		-	-	-
Olive oil, g	10.0		-	-	-
Span 20, g	6.0		-	-	-
Tween 20, g	6.0		-	-	-
Vaseline, g	-		-	ad 100.0	-
PIONIER^®^ PLW, g	-		-	-	ad 100.0
Water, g	ad 100.0		ad 100.0	-	-
Content, g	100.0 ± 0.5		100.0 ± 0.5	100.0 ± 0.5	100.0 ± 0.5

**Table 2:** Correlation between individual phenolic compounds penetrated into the skin layers and the antioxidant activity detected in these skin layers.

		Chlorogenic Acid	Rutin	Quercetin
		Epidermis		
Antioxidant activity	Pearson correlation	0.688	0.500	0.729
	Sig. (2-tailed)	0.200	0.391	0.162
		Dermis		
Antioxidant activity	Pearson correlation	0.765	0.824	0.843
	Sig. (2-tailed)	0.132	0.087	0.073

Pearson correlation coefficients: 0 < |r| ≤ 0.3 is a weak correlation; 0.3 < |r| ≤ 0.7 is a moderate correlation; 0.7 < |r| ≤ 1 is a strong correlation [63].

## Data Availability

All datasets generated for this study are included in the article.
